# LncRNA LINC00472 regulates cell stiffness and inhibits the migration and invasion of lung adenocarcinoma by binding to YBX1

**DOI:** 10.1038/s41419-020-03147-9

**Published:** 2020-11-03

**Authors:** Xiangying Deng, Wei Xiong, Xianjie Jiang, Shanshan Zhang, Zheng Li, Yanhong Zhou, Bo Xiang, Ming Zhou, Xiaoling Li, Guiyuan Li, Zhaoyang Zeng, Zhaojian Gong

**Affiliations:** 1 Department of Oral and Maxillofacial Surgery, The Second Xiangya Hospital, Central South University, Changsha, Hunan 410011 China; 2 NHC Key Laboratory of Carcinogenesis and Key Laboratory of Carcinogenesis and Cancer Invasion of the Chinese Ministry of Education, Cancer Research Institute, Central South University, Changsha, Hunan China; 3 Hunan Key Laboratory of Nonresolving Inflammation and Cancer, Disease Genome Research Center, the Third Xiangya Hospital, Central South University, Changsha, Hunan China; 4 Department of Stomatology, Xiangya Hospital, Central South University, Changsha, Hunan China

**Keywords:** Cancer imaging, Non-small-cell lung cancer, Cell invasion, Biomarkers

## Abstract

There is increasing evidence that long non-coding RNAs (lncRNAs) play important roles in human tumorigenesis. By using publicly available expression profiling data from lung adenocarcinoma and integrating bioinformatics analysis, we screened a lncRNA, LINC00472. LINC00472 expression in lung adenocarcinoma tissues was significantly lower and tightly associated with patient prognosis and TNM clinical stages in lung adenocarcinoma. LINC00472 also inhibited lung adenocarcinoma cell migration and invasion and increased cell stiffness and adhesion. RNA pull down and RIP assays identified that LINC00472 interacted with the transcription factor Y-box binding protein 1 (YBX1), which partially reversed the inhibition of cell migration and invasion and increased LINC00472-induced cell stiffness and adhesion. LINC00472 also regulated the density and integrity of F-actin in A549 and PC-9 cells possibly via YBX1. LINC00472 inhibited the cell epithelial-mesenchymal transition (EMT) processes via the modulation of YBX1. These results indicated that LINC00472 inhibited the cell EMT process by binding to YBX1, and affected the mechanical properties of the cell, ultimately inhibited its ability to invade and metastasize. Collectively, the present study provides the first evidence that LINC00472 changes the mechanical properties and inhibits the invasion and metastasis of lung adenocarcinoma cells.

## Introduction

Lung cancer is one of the leading causes of cancer-related death worldwide^1,2^. One of the most common types of lung cancer is non-small cell lung cancer (NSCLC). Lung adenocarcinoma is a subtype of NSCLC, and it accounts for ~50% of all NSCLCs^3,4^. Chemotherapy and molecular-targeting therapy for NSCLC have made great progress, but its overall 5-year survival rate is <15%^5^. Therefore, it is important to understand the underlying mechanisms that regulate NSCLC pathogenesis and identify effective therapies for NSCLC patients.

LncRNAs are a class of regulatory non-coding RNAs (ncRNAs) that are typically over 200 nt in length and exhibit limited protein-coding capacity^6–8^. Tens of thousands of lncRNAs may be encoded in the human genome^9,10^, but their exact roles remain elusive. LncRNAs have attracted attention for their new role in cancer^11,12^. Increasing studies showed that lncRNAs are involved in cancer progress via regulation of the occurrence of the EMT^13,14^, which is related to the biophysical characteristics of cells^15^.

Recent studies showed that cellular mechanical properties are markers of a variety of cellular processes, including malignant transformation, migration, and the apoptosis of cancer cells^16–20^. The morphology, mechanical properties and composition of the extracellular matrix (ECM) play key roles in the fate of cells^21^. When cells are in the process of carcinogenesis and stimulated by the outside world, their physical properties, such as morphology, elasticity and adhesion, change. Therefore, integrating information from multiple individual factors, such as cell adhesion, roughness and stiffness, provides new insights into a more accurate understanding and prediction of cellular behavior.

The present study analyzed GEO and TCGA data and found that LINC00472 expression was significantly lower in lung adenocarcinoma tissues, and it was related to the clinical outcome of lung adenocarcinoma patients. In vitro experiments indicated that LINC00472 inhibited the migration and invasion of lung adenocarcinoma cells and increased cell stiffness and adhesion. These results suggest that LINC00472 plays a critical role in lung adenocarcinoma progression and prognosis, and it may be used as a potential diagnostic and prognostic biomarker.

## Materials and methods

### Data analysis

The lung adenocarcinoma gene expression GEO dataset (GSE27262 and GSE31210) were downloaded from the GEO database^22–25^. Significant Analysis of Microarray (SAM) software was used to analyze differentially expressed lncRNAs between normal lung epithelium tissues and lung adenocarcinoma tissue samples in the two GEO datasets (GSE27262 and GSE31210), respectively. The cut-off value for differentially expressed lncRNA was set to a >1.5-fold difference, and the false discovery ratio (FDR) was <0.05.

### Cell culture, transfection, and plasmids

Lung adenocarcinoma A549 and PC-9 cell lines, and human normal lung epithelial cells (BEAS-2B) were purchased from ATCC and cultured in RPMI 1640 medium supplemented with 10% fetal bovine serum (FBS, Biological Industries, Israel) and a penicillin-streptomycin solution (100 U/ml penicillin, 100 µg/ml streptomycin) (FBS, Biological Industries, Israel) at 37 °C in a humidified CO_2_ (5%) incubator. A LINC00472 transcript (9566 bp, NR_121612.1) was inserted in the pcDNA3.1(+) plasmid by TSINGKE Biological Technology (Changsha, China). The pCMV–N-HA-YBX1 recombinant vector and pcDNA3.1(+)-TGF-β1 recombinant vector was constructed by our research group. For the plasmid transfection, the Lipofectamine 3000 Transfection Reagent Kit (Life Technologies) was used with OptiMEM medium (Invitrogen).

### RNA extraction and qRT-PCR

Total RNA was extracted from cells using TRIzol reagent (Ambion, Cat.207008, USA) according to the manufacturer’s instructions. For lncRNA and mRNA quantification, RNA was reverse transcribed into cDNA using a reverse transcription kit (abm, cat#G492, Canada). Quantitative real-time PCR was performed using cDNA primers specific for the lncRNA. qRT-PCR was performed in a CFX96 real-time PCR detection system (Bio-Rad, Hercules, CA, USA) using an EvaGreen Kit (abm, Canada) to determine the relative expression levels of the target genes. The primer sequences are listed in Supplementary Table 1.

### Transwell invasion assay and wound scratch assay

Transwell invasion assays were performed using 8.0-μm Transwell permeable supports (Millipore, Cat.MCEP24H48, USA). Cells were harvested 24 h after transfection and treated with pemetrexed (Selleck, Cat.S1135, USA). A549 cells were treated at a concentration of 4.8 μM/L for 24 h, and PC-9 cells were treated at a concentration of 9.6 μM/L for 24 h. Cells (2 × 10^5^) suspended in 200 μl of serum-free medium were seeded into the upper chamber precoated with Matrigel Matrix (BD Biosciences), and 800 μl of medium containing 20% FBS was added to the lower chamber. After incubation for 48 h, cells that did not invade through the membrane were mechanically removed using a cotton swab. Paraformaldehyde (4%) was used to fix the cells on the bottom surface of the membrane for 10 min, and the cells were stained with a 0.4% crystal violet solution. The invading cells were imaged using digital microscopy (Nikon). The number of invasive tumor cells was counted from five randomly selected 20× fields for each experiment and averaged.

For the wound scratch assay, cells were transfected after 24 h and treated with pemetrexed. A549 cells were treated at a concentration of 4.8 μM/L for 24 h and PC-9 cells were treated at a concentration of 9.6 μM/L for 24 h. Cells were grown to 90% confluence in 6-well culture plates. A p200 pipet tip was used to create a scratch in the cell monolayer. Images were captured using digital microscopy at the indicated times. The wound width was measured using an ocular ruler to ensure that all wounds were the same width at the beginning of each experiment.

### Measurement of cellular biophysical properties using atomic force microscopy (AFM)

A JPK NanoWizard 4 BioScience AFM (JPK Instruments, Berlin, Germany) was used to optically align the probe to the cells. The probes used in this study were HYDRA6V-100NG (AppNano, CA, USA) with a nominal spring constant of 0.292 N/m. During the indentation process, the loading and retraction speeds of all experiments were maintained at ~2.5 μm/s to avoid viscosity effects. Measurements were made in PBS at room temperature, and the cells were plated on the bottom of the cell culture dish. After transfection of the LINC00472 overexpression vector for 24 h, cells were washed twice with PBS, fixed with 2% glutaraldehyde for 45 s and a 4% polymethanol solution for 20 min. Cells were washed five or more times with PBS and maintained in an appropriate amount of PBS for subsequent AFM scanning. The indentation depth was at least 1 mm to better simulate physiologically occurring deformations. Imaging was performed using the QI mode, and images of the AFM scan were analyzed using JPK image processing software. The force and indentation curves from each measurement were analyzed using a Hertz model to obtain the Young’s modulus for each cell.

### Western blotting

Lysis, electrophoresis, and target protein visualization were performed as described previously^26^. In brief, 30 μg of cell lysates were separated using 10% sodium dodecyl sulfate-polyacrylamide gel electrophoresis (SDS-PAGE) and transferred onto a PVDF membrane. Membranes were incubated overnight at 4 °C with primary anti-Vimentin (1:1000, Cell Signaling Technology, CST), E-cadherin (1:1000, CST), N-cadherin (1:1000, CST), Snail (1:500, CST), and YBX1 (1:500, protein-tech, China) antibodies. The blots were washed with phosphate-buffered saline Tween-20 (PBST) and incubated with a horseradish peroxidase-conjugated secondary antibody for 2 h at room temperature. The signal was visualized using an ECL detection reagent (Millipore, Cat.WBKLS0500, USA) and quantified using densitometry in ImageJ software (http://rsb.info.nih.gov/ij). GAPDH was used as a loading control and was detected using a goat anti-GAPDH antibody (Cell Signaling Technology, UK).

### RNA immunoprecipitation (RIP)

RIP assays were performed using a Magna RIP kit (Cat. 17–701, Millipore, USA) according to the manufacturer’s instructions. A YBX1 antibody (Protein-tech, China) was used for the RIP assay. IgG was used as a negative control, and SNRNP70 was used as a positive control.

### In vitro transcription assays and RNA pull-down assays

In vitro translation assays were performed using T7 RNA polymerase according to the manufacturer’s instructions (Promega, USA). LINC00472 RNAs were labeled using a Biotin RNA Labeling Mix (Roche, Germany). RNA pull-down assays were performed with Dynabeads^TM^ M-280 streptavidin (Thermo, USA). The lncRNA-interacting proteins were eluted and subjected to Western blot analysis.

### Immunofluorescence

The cells were fixed with 4% paraformaldehyde at room temperature for 20 min and washed three times with phosphate-buffered saline (PBS) for 5 min each. The cells were blocked in PBS containing 7% FBS for 30 min. Cells were incubated with Alexa Fluor 488 phalloidin (Sigma, USA) at 37 °C for 60 min. Cells were incubated with anti-Snail (1:200), E-cadherin (1:200), N-cadherin (1:200) and Vimentin (1:200) antibodies at 4 °C for overnight. Cells were washed three times with PBS, then incubated with secondary antibody (Abcam, Goat polyclonal Secondary Antibody to Rabbit IgG, ab150077) for 60 min at 37 °C. Cells were washed three times with PBS, then incubated with 4,6-diamidino-2-phenylindole (DAPI) for 10 min at room temperature, and washed three times with PBS. Immunofluorescence images were collected using a confocal fluorescence microscope (UltraViewVox; PerkinElmer, Waltham, MA, USA) and quantified using densitometry in ImageJ software (http://rsb.info.nih.gov/ij).

### Statistical analysis

Statistical analyses were performed using GraphPad Prism 7 software. Student’s *t*-tests were used to evaluate significant differences between any two groups of data for multiple comparisons. Overall survival (OS) or relapse-free survival (RFS) were calculated using the Kaplan–Meier method, and the results of the analyses were considered significant in a log-rank test when *p* < 0.05. All data are presented as the means ± standard deviation (SD). Differences were considered significant when *p* < 0.05. Data from the experiments are expressed as the means ± SD from at least three independent experiments.

## Results

### LINC00472 exhibits low expression in lung adenocarcinoma and correlates with patient prognosis

**Fig. 1:**
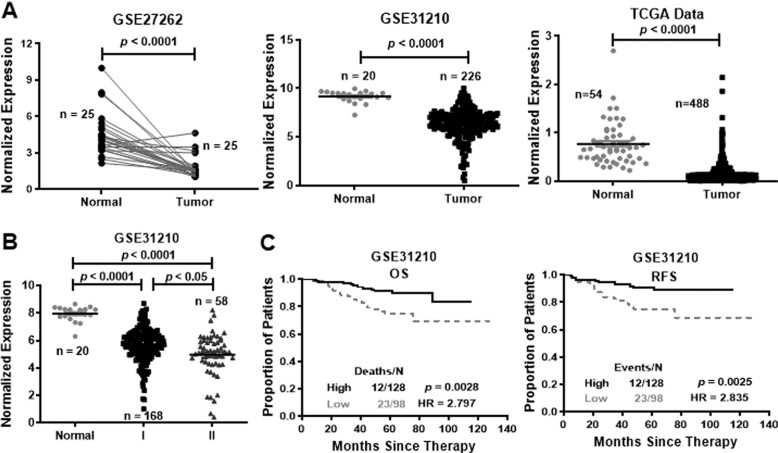
LINC00472 is lowly expressed in lung adenocarcinoma tissues and associated with clinical parameters. **a** LINC00472 is lowly expressed in lung adenocarcinoma tissues compared with noncancerous lung tissues analyzed using the GEO database (GSE27262 and GSE31210) and the TCGA database. **b** The expression of LINC00472 is associated with the clinical-pathological stage (Normal: normal tissues; I, II: clinical stages I, II). LINC00472 expression was significantly lower in patients with advanced TNM stage according to the GSE31210 data. **c** Overall survival (OS) and relapse-free survival (RFS) analysis of patients were performed according to the expression of LINC00472 using a Kaplan–Meier curve. Kaplan–Meier survival plots demonstrate that low LINC00472 expression levels are correlated with worse OS and RFS in lung adenocarcinoma patients.

SAM microarray analysis software identified 3658 differentially expressed probes in GSE31210 and 6172 differentially expressed probes in GSE27262. Analysis of these probes revealed that 1995 probes were differentially expressed in both groups: 403 were highly expressed and 1592 were lowly expressed in lung cancers (Supplementary Fig. 1a). Based on NetAffx, Refseq and Ensembl non-coding RNA annotations, we identified 14 overlapping probes representing 13 lncRNAs, including 1 highly expressed lncRNA and 12 lowly expressed lncRNAs in lung cancer samples (Supplementary Fig. 1b, c). Among the 12 lowly expressed lncRNAs, one probe, LINC00472, attracted our attention. LINC00472 expression was also low in lung adenocarcinoma tissues compared to noncancerous lung epithelium tissues according to the GEO database (GSE27262 and GSE31210) (Fig. 1a). The TCGA data also showed that LINC00472 expression was significantly lower in lung adenocarcinoma tissues (Fig. 1a).

Further analyses showed that the low expression of LINC00472 was associated with clinical parameters. The results showed that the LINC00472 level was associated with the TNM stage in the GSE31210 dataset. Patients with lower LINC00472 expression were associated with an advanced TNM stage (Fig. 1b). Kaplan–Meier analysis indicated that lower LINC00472 expression correlated with worse OS and RFS in the GSE31210 database (Fig. 1c).

### Overexpression of LINC00472 inhibits lung adenocarcinoma cell migration and invasion

**Fig. 2:**
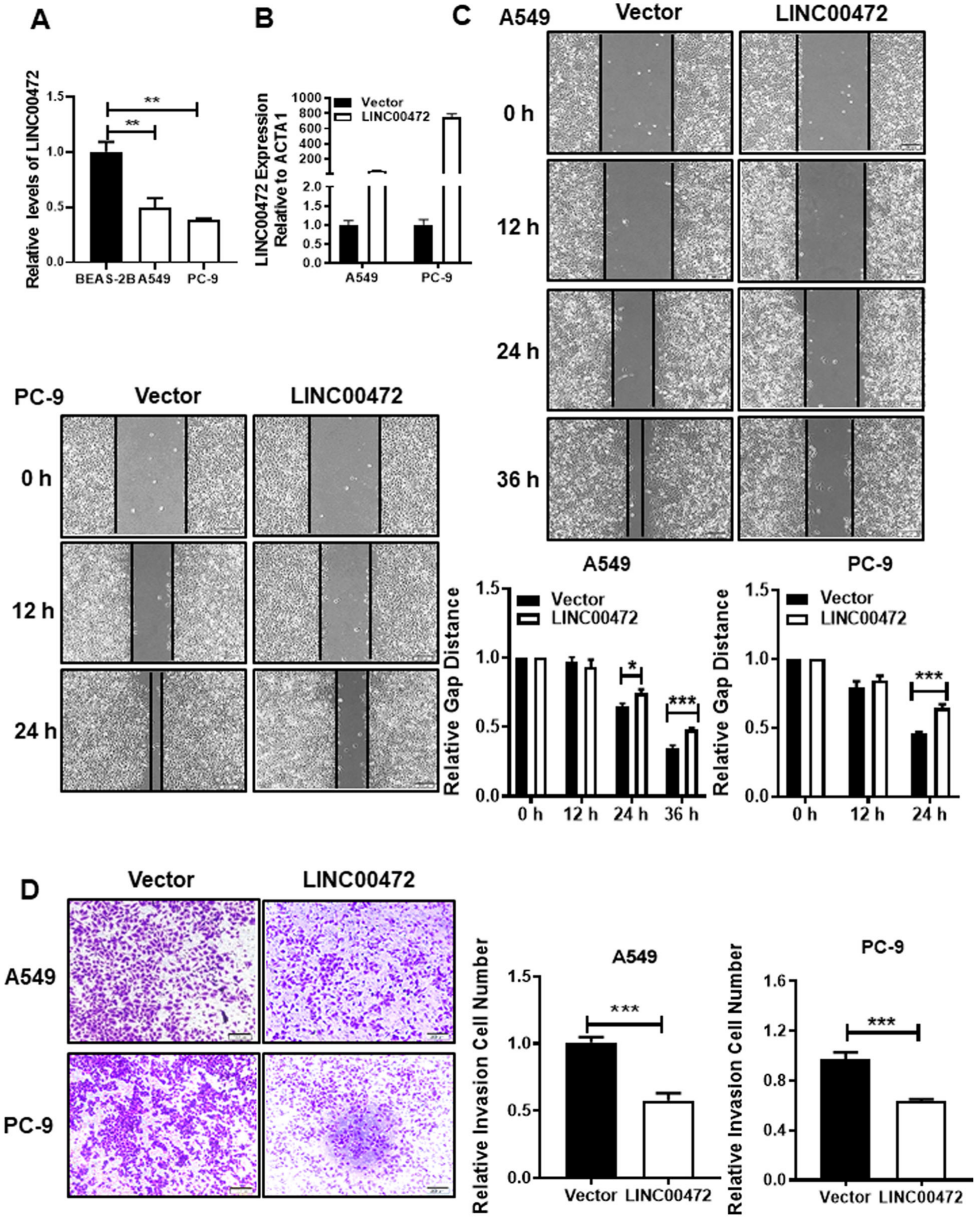
Overexpression of LINC00472 inhibits migration and invasion of cells. **a** The expression levels of LINC00472 in lung adenocarcinoma cell lines and human lung epithelial cell line BEAS-2B. **b** The overexpression efficiency of LINC00472 in A549 and PC-9 cells were detected using qRT-PCR. **c** Overexpression of LINC00472 significantly inhibits migration of A549 and PC-9 cells. **d** Overexpression of LINC00472 significantly inhibits the invasion of A549 and PC-9 cells. The above chart summarizes data from three separate experiments. Vector: the empty vector; LINC00472: the LINC00472 overexpression vector. *p < 0.05; **p < 0.01; ***p < 0.001.

To further explore the role of LINC00472 in the development of lung adenocarcinoma, we selected the A549 and PC-9 cell lines for the following experiments. The results showed a lower expression of LINC00472 in A549 and PC-9 tumor cells compared to the human normal lung epithelial cells BEAS-2B (Fig. 2a). As shown in Fig. 2b, LINC00472 was successfully overexpressed in the A549 and PC-9 cells. The results of the scratch test showed that the migration abilities of A549 and PC-9 cells transfected with the LINC00472 overexpressed vector were significantly weaker than the empty vector (Fig. 2c). Invasion assay results showed that A549 and PC-9 cells transfected with the overexpressing LINC00472 vector had a weaker invasive ability compared to cells transfected with the empty vector (Fig. 2d). These results indicated that LINC00472 inhibited cell migration and invasion.

### Overexpression of LINC00472 alters the biophysical properties of cells

**Fig. 3:**
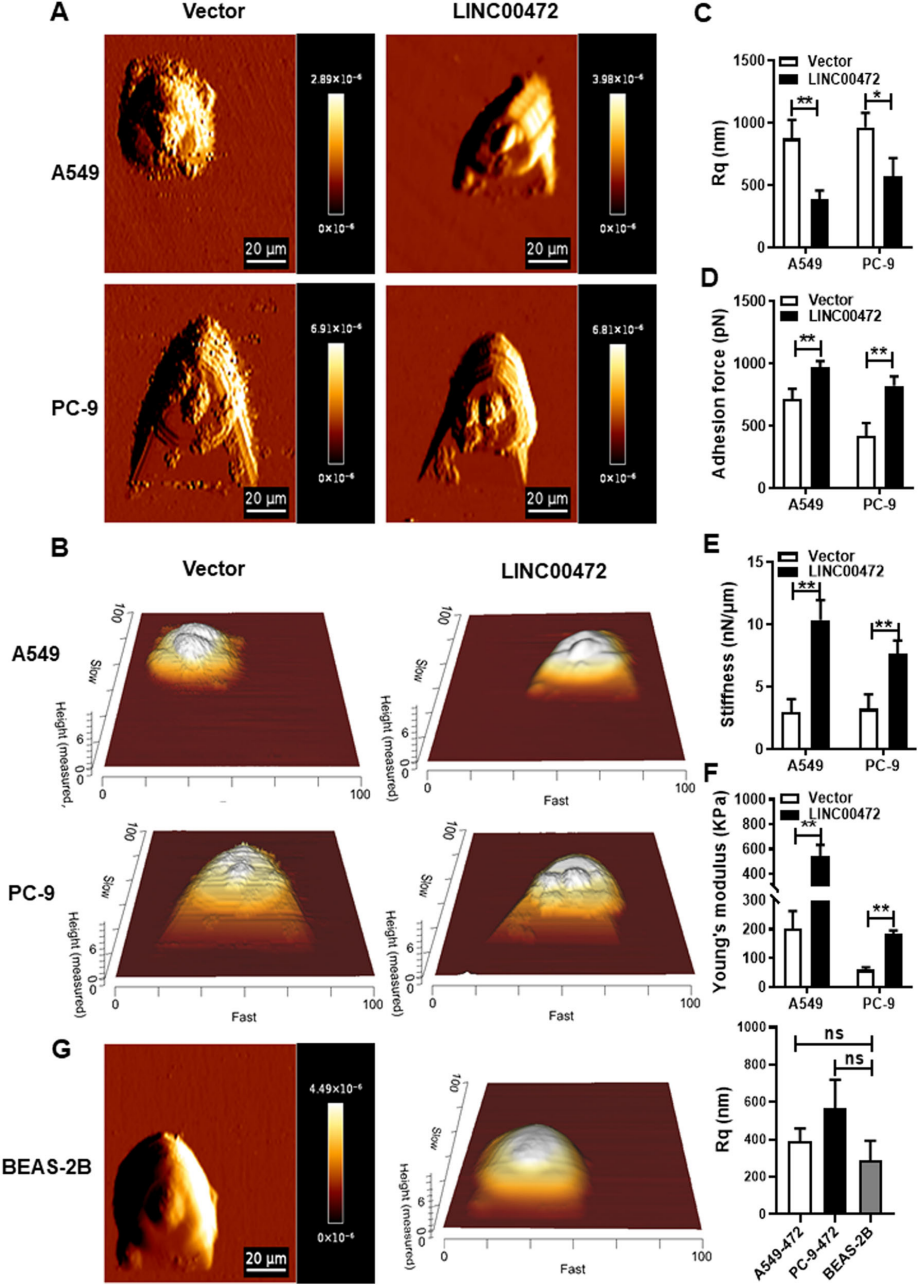
Overexpression of LINC00472 alters the surface morphology and mechanical properties of cells. **a** Representative AFM deflection images of A549 and PC-9 cells transfected with the LINC00472 overexpression vector. **b** 3D distribution of the cell heights of A549 and PC-9 cells transfected with the LINC00472 overexpression vector. **c** LINC00472 affects the surface morphology (Rq) of A549 and PC-9 cells. **d** Analysis of the adhesion force of the A549 and PC-9 cells. **e** Analysis of the stiffness of A549 and PC-9 cells. **f** Analysis of the Young’s modulus of A549 and PC-9 cells. **g** Representative AFM deflection images of BEAS-2B, 3D distribution of the cell heights of BEAS-2B, and analysis of surface morphology (Rq) of BEAS-2B cells. The force curve was used to calculate the mechanical properties. Data on adhesion, stiffness, Rq and Young’s modulus are based on analyses of the JPK image processing software. Vector: the empty vector; LINC00472: the LINC00472 overexpression vector. *p < 0.05; **p < 0.01.

The mechanical properties of cells determine cell movement. Therefore, the mechanical properties of cells also affect the migration and invasion of cancer cells^27^. Recent studies found that the biophysical properties of cells change during carcinogenesis^28^. Therefore, we analyzed the biophysical properties of A549 and PC-9 cells transfected with the LINC00472 overexpression vector using AFM (Fig. 3a, b). The Rq value of the cell surface decreased, and the cell surfaces became smoother and closer to the morphology of normal cells (Fig. 3g) when transfected with the LINC00472 overexpression vector in A549 and PC-9 cells (Fig. 3c). The adhesion increased, which led to a reduction in cell migration (Fig. 3d). The stiffness and Young’s modulus of the cells also increased in LINC00472-transfected cells, which suggests that cells became stiffer and their ability to deform decreased, which led to reduced cell invasiveness (Fig. 3e, f). These results suggest that LINC00472 alters the mechanical properties of lung adenocarcinoma cells, which affects the physiological activities of cells, including cell migration and invasion.

### Interaction of LINC00472 with YBX1

**Fig. 4:**
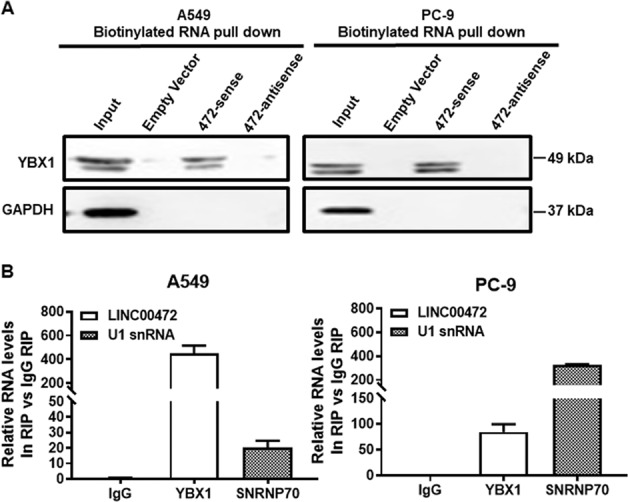
The interaction between LINC00472 and YBX1. **a** RNA pull-down assays and Western blot assays show that biotinylation of LINC00472 could bind YBX1 in A549 and PC-9 cells. **b** The RNA immune precipitation (RIP) experiment was performed by using YBX1, SNRNP70, and IgG antibodies to probe A549 and PC-9 cell extracts, and the level of the coprecipitated RNAs were determined using qRT-PCR.

To explore the possible mechanism underlying the biological function of LINC00472, we used website predictions (http://pridb.gdcb.iastate.edu/RPISeq/ index.html) to identify potential combinations of LINC00472. The prediction showed an interaction between LINC00472 and YBX1. To confirm this interaction, we performed an RNA pull-down assay and found that LINC00472 specifically bound to the YBX1 protein using Western blotting (Fig. 4a). A RIP assay using a YBX1-specific antibody was also performed to confirm whether the YBX1 specifically bound to LINC00472 (Fig. 4b). As expected, LINC00472 was enriched in the anti-YBX1 group compared to the control IgG group. These results indicated that LINC00472 bound to the YBX1 protein.

### Overexpression of YBX1 partially reverses the cell phenotype caused by LINC00472

**Fig. 5:**
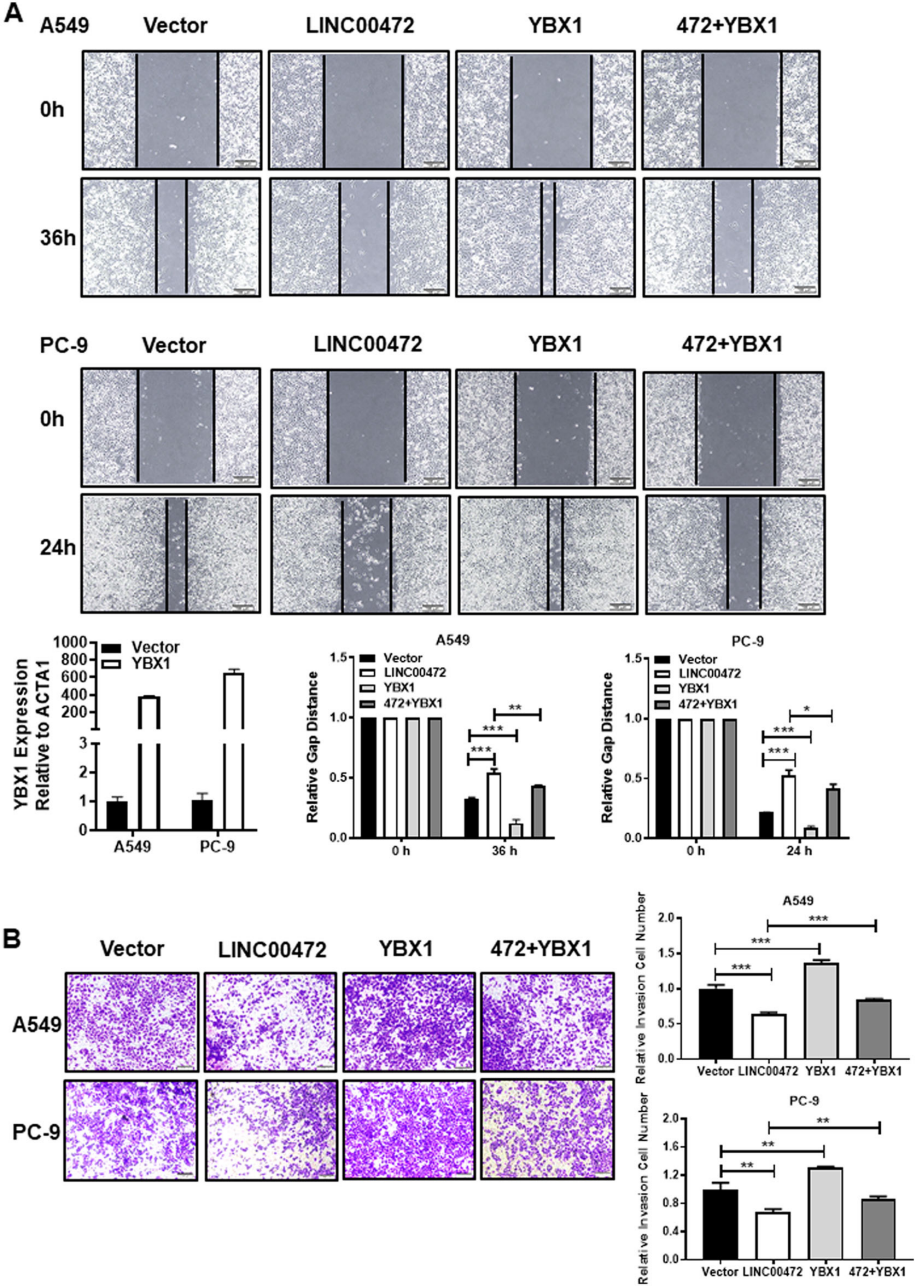
Overexpression of YBX1 partially reverses the function of LINC00472. A549 and PC-9 cells were transfected with empty vector, LINC00472 or YBX1 overexpression plasmid and cotransfected with LINC00472 and YBX1 overexpression plasmid. **a**–**b** After cotransfected with LINC00472 and YBX1 overexpression vector, the overexpression efficiencies of YBX1 in the A549 and PC-9 cells were measured by qRT-PCR. Overexpression of YBX1 relieved the inhibit of cell migration and invasion caused by LINC00472 overexpression. Vector: the empty vector; LINC00472: the LINC00472 overexpression vector; YBX1: the YBX1 overexpression vector. *p < 0.05; **p < 0.01; ***p < 0.001.

**Fig. 6:**
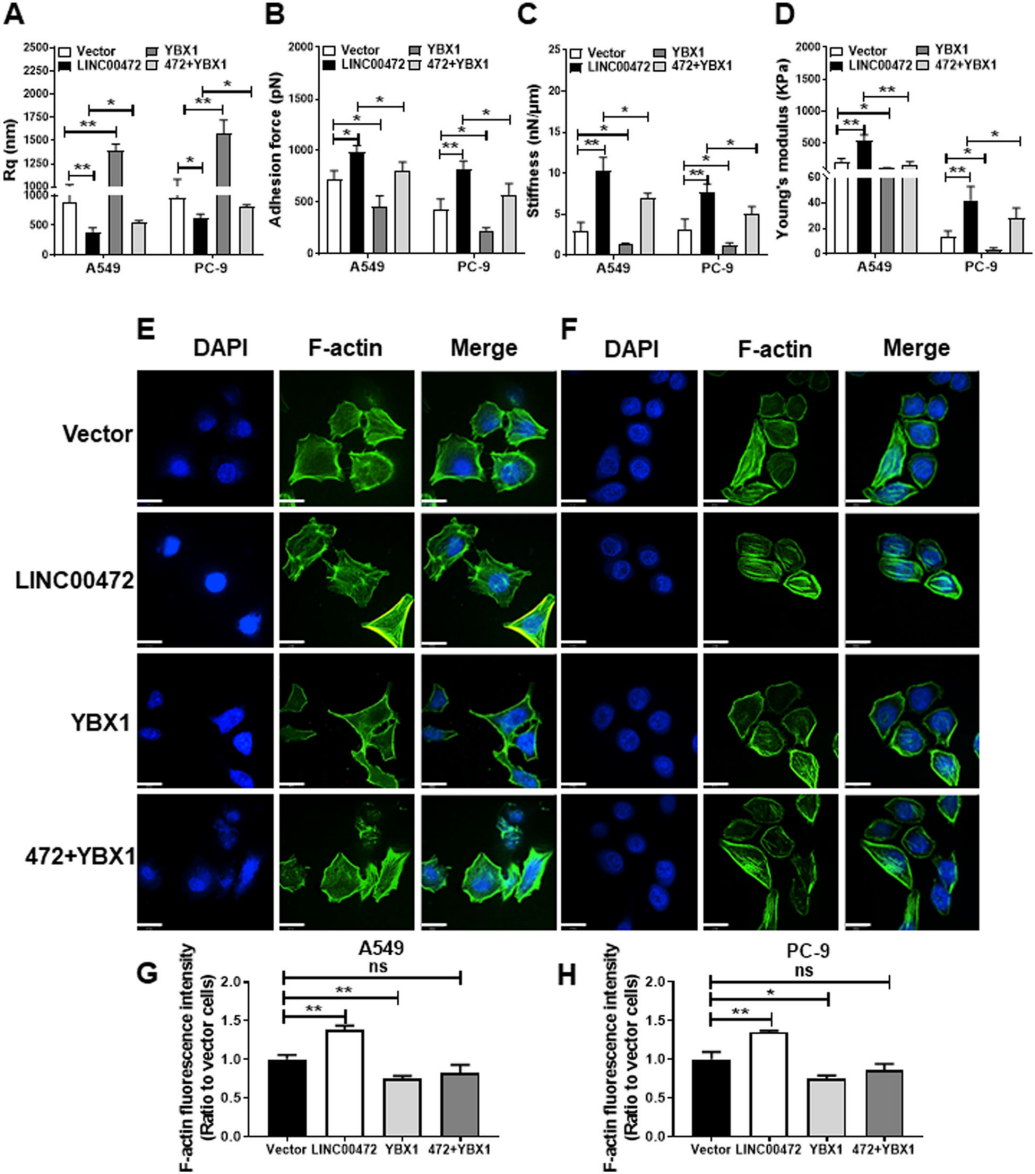
Overexpression of YBX1 partially reverses the function of LINC00472 on the cellular biophysical properties and F-actin. **a** YBX1 participates in the effects of LINC00472 on the surface morphology (Rq) of A549 and PC-9 cells. **b** YBX1 reverses the effects of LINC00472 on the adhesion force of A549 and PC-9 cells. **c** YBX1 affects the function of LINC00472 on the stiffness of A549 and PC-9 cells. **d** YBX1 modulates the effects of LINC00472 on the Young’s modulus of A549 and PC-9 cells. **e** Detection of changes in F-actin in A549 cells after transfection or cotransfection with the LINC00472 overexpression vector or the YBX1 overexpression vector. **f** Detection of changes in F-actin in PC-9 cells after transfection or cotransfection with the LINC00472 overexpression vector or the YBX1 overexpression vector. Scale bar = 19 μm. **g** Statistics of fluorescence intensity in A549 cells after the transfection of different vectors. **h** Statistics of fluorescence intensity in PC-9 cells after the transfection of different vectors. Vector: empty vector; LINC00472: LINC00472 overexpression vector; YBX1: YBX1 overexpression vector. *p < 0.05; **p < 0.01.

To identify whether YBX1 participated in the function of LINC00472 during lung adenocarcinoma migration and invasion, the LINC00472 overexpression vector and a YBX1 overexpression vector were cotransfected into cells. The data showed that overexpression of YBX1 partially reversed the inhibition of cell migration and invasion due to the overexpression of LINC00472 (Fig. 5). The overexpression of YBX1 partially reversed the LINC00472 overexpression-induced changes in the cell biophysical properties (Fig. 6a–d, Supplementary Fig. 2). In contrast to the vector group, F-actin in the overexpression LINC00472 group was denser and packed in a regular arrangement. F-actin in the YBX1 overexpression group was less sparse, and the arrangement was more disordered with a lack of integrity (Fig. 6e–h). The density and integrity of F-actin in the overexpression LINC00472 and YBX1 groups were similar to the vector group (Fig. 6e–h). These results showed that LINC00472 enhanced the integrity and density of F-actin, and YBX1 caused the loss of integrity of F-actin in A549 and PC-9 cells. Therefore, LINC00472 regulate the density and integrity of F-actin possibly via binding to YBX1. These results indicated that LINC00472 mediates lung adenocarcinoma tumorigenesis by binding to YBX1.

### LINC00472 regulates the EMT pathway via YBX1

**Fig. 7:**
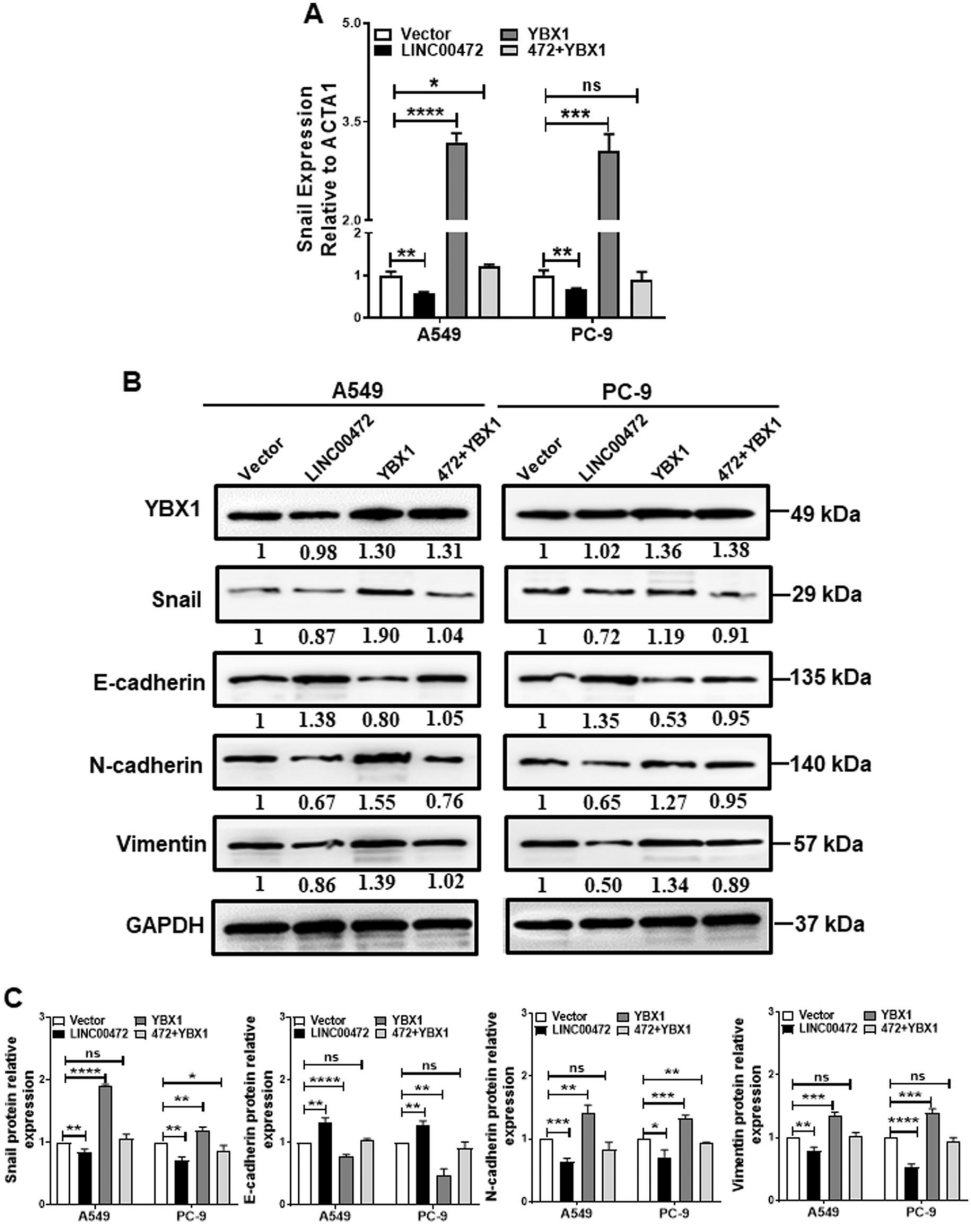
LINC00472 regulates the EMT processes through YBX1. **a** The mRNA expression of Snail was measured in A549 and PC-9 cells after transfection or cotransfection with the LINC00472 overexpression vector or the YBX1 overexpression vector. **b** The protein expression of Snail, Vimentin, E-cadherin and N-cadherin were measured in A549 and PC-9 cells after transfection or cotransfection with the LINC00472 overexpression vector or the YBX1 overexpression vector. The vector was used as a control and standardized to 1, and the number below the band is the ratio of target band/vector band for grayscale scanning. **c** Statistical analysis of gray values of three Western blotting results. Vector: empty vector; LINC00472: LINC00472 overexpression vector; YBX1: YBX1 overexpression vector. *p < 0.05; **p < 0.01; ***p < 0.001; ****p < 0.0001, ns: no significance.

YBX1 acts as a cancer-promoting gene in a variety of cancers^29,30^. YBX1 also activates the translation of Snail mRNA to directly induce EMT^31^. Previous studies showed that the cell stiffness changed during the EMT process^15^. To investigate whether the interaction between LINC00472 and YBX1 affected the regulation of Snail, we performed qRT-PCR, western blot and immunofluorescence assays. As shown in Fig. 7a, exogenous LINC00472 expression slightly decreased the mRNA levels of Snail. We further found that exogenous LINC00472 expression decreased the protein levels of Snail, N-cadherin and vimentin and upregulated the protein level of E-cadherin (Fig. 7b, c, Supplementary Fig. 3). However, exogenous YBX1 expression upregulated the protein levels of Snail, N-cadherin and vimentin and decreased the protein level of E-cadherin (Fig. 7b, c, Supplementary Fig. 3). The overexpression of YBX1 attenuated the effects of LINC00472 overexpression on the protein levels of Snail, E-cadherin, vimentin, and N-cadherin (Fig. 7b, c, Supplementary Fig. 3). Collectively, these findings revealed that LINC00472 inhibited Snail translation and EMT processes by modulating YBX1 in the cells.

## Discussion

The present study screened the lncRNA LINC00472 in lung adenocarcinoma, and its expression was associated with RFS and OS in lung adenocarcinoma patients. Although many studies showed that LINC00472 played a suppressor gene role in breast cancer, liver cancer and colorectal cancer, including inhibition of the proliferation, migration and invasion of cancer cells and promotion of apoptosis^32–34^, the present study is the first time that LINC00472 was associated with cancer cellular mechanical properties in lung cancer.

AFM is a powerful tool that is used to obtain cell surface topography and specific information about cellular mechanical properties^35^. It is widely used in biomedical research because the disease often leads to changes in cell morphology and mechanical properties^36^. During the invasion and metastasis of cancer cells, cell-to-cell adhesion is generally weakened, and the shape and stiffness of the cells also change^37^. The present study found that LINC00472 attenuated cell migration by increasing cell–cell adhesion and inhibited cell invasion by increasing cell stiffness. The reduced surface roughness of cancer cells indicated that these cells tended toward normal epithelial cells.

There are no reports on the relationship between lncRNAs and the mechanical properties of cancer cells. However, many studies reported that lncRNAs affect the EMT pathway of cancer cells^38,39^. During EMT, the phenotype of epithelial cells gradually disappears, and the phenotype of interstitial cells is gradually acquired, including the downregulation of epithelial-specific proteins and the upregulation of stromal cell-specific proteins, which increase cell processes and cell motility^40^. These changes may affect the surface topography and mechanical properties of the cells. Previous studies showed that the stiffness of the cells changes when the cells undergo EMT^15^. TGF-β is a key factor in the induction of EMT in tumor cells^41^. We used TGF-β1 to induce EMT in A549 and PC-9 cells and used AFM to detect the biophysical properties of the cells. We found that the surfaces of TGF-β1-induced A549 and PC-9 cells became rougher, less adhesive, and less stiff, which indicates that the biophysical properties of lung adenocarcinoma cells were altered during the induction of EMT. Compared with the TGF-β1-induced EMT group, we found that roughness was partially inhibited; adhesion, stiffness and Young’s modulus were partially promoted after simultaneous overexpression of LINC00472 and TGF-β1 in cells. (Supplementary Fig. 4). This result further demonstrates that LINC00472 changes the biophysical properties of cells and inhibits of the cellular EMT processes and ultimately inhibits lung adenocarcinoma cell invasion and metastasis. Many studies also showed that YBX1 was involved in the mechanism of lncRNAs^3,42,43^ and influenced cancer cell migration and invasion by directly activating Snail translation^44^ and inducing EMT^31^. Therefore, we presumed that LINC00472 would perform biological functions in a similar manner. We found that the interaction of LINC00472 with YBX1 slightly inhibited Snail translation and cellular EMT processes. The mechanism may be that LINC00472 binding to YBX1 resulted in a decrease in the amount of free YBX1 that is used to activate the translation of Snail mRNA, which decreased Snail expression and inhibited the EMT of the cells. However, LINC00472 simply bound YBX1 and had no effect on its expression in this process. Therefore, the overexpression of LINC00472 did not alter the expression of YBX1 in cells. Changes in the expression of EMT marker proteins likely change the cell skeletal structure and adhesion characteristics, which result in changes in cell mechanical properties and affect the physiological activities of cancer cells.

**Fig. 8:**
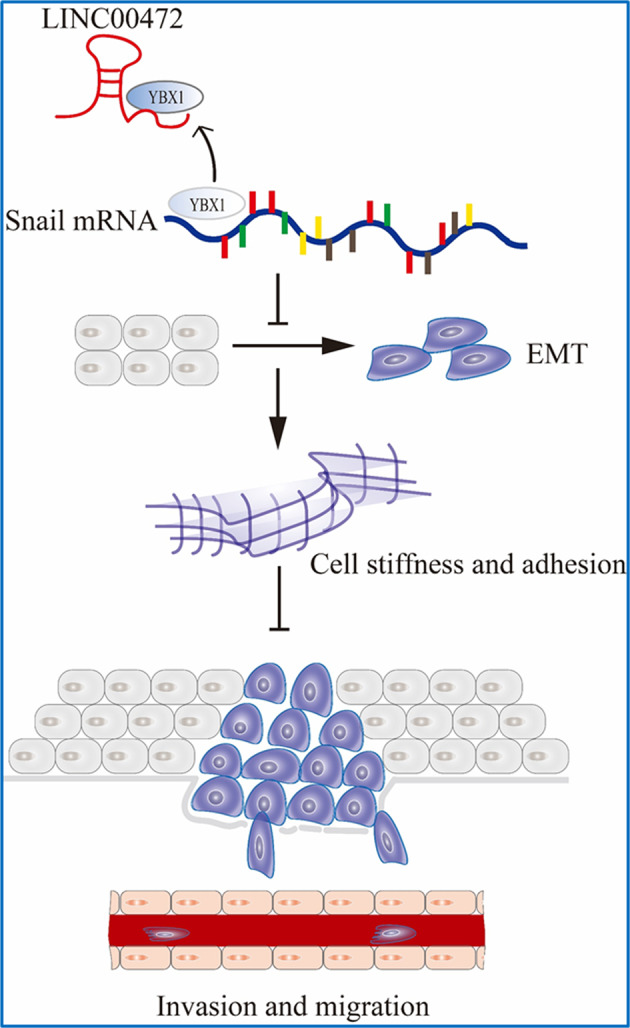
Schematic diagram of the effects of LINC00472 on the migration and invasion of lung adenocarcinoma. The interaction between LINC00472 and YBX1 slightly downregulated Snail expression and inhibited the EMT process. The expression changes of EMT-related markers potentially enhance the cells stiffness and adhesion and ultimately inhibit the invasion and metastasis of cancer cells.

Vimentin, E-cadherin and F-actin are associated with cell biophysical properties. Vimentin and F-actin play key roles in maintaining cellular morphology and mechanics^45–49^ and may remodel the cytoskeleton and alter cell stiffness and affect the invasion and migration ability of cells. The cytoskeleton is related to the coregulation of multiple molecules rather than the action of a single molecule. Therefore, we hypothesize that vimentin and F-actin work together to regulate the cytoskeleton via YBX1 and affect cell stiffness and its ability to metastasize and invade. After overexpression LINC00472 in A549 and PC-9 cells, vimentin expression decreased, F-actin became denser and more regular, and cell stiffness increased, which suggests that LINC00472 inhibits the metastasis of lung adenocarcinoma by altering the expression of vimentin and the density and integrity of F-actin. E-cadherin is related to cell adhesion^50–52^, and its expression increased, which indicates that the adhesion between cells and cells and the ECM increases and cell motility decreases. The specific mechanisms will be further explored in subsequent studies. The combining of LINC00472 with YBX1 inhibited cellular EMT process and affected the mechanical properties of the cell, which may explain the decline in the ability of cancer cells to invade and metastasize. A schematic diagram of the effects of LINC00472 on the migration and invasion of lung adenocarcinoma is shown in Fig. 8. LINC00472 may be used as a biomarker^53^.

Biomarkers for diagnosis and treatment are not limited to a single molecule but may be due to changes in the biophysical properties or state of the cells caused by a molecule^54^. Many studies showed that cell mechanics are a promising biomarker for cell or tissue status^55,56^. AFM distinguishes between cancer cells and noncancerous cells via the detection of the Young’s modulus of cells, i.e., cell stiffness. Therefore, detecting the stiffness of a cell may reflect its physiological state. AFM indentation and traction analyses in the present study showed that the cancer cells overexpressing LINC00472 had a higher hardness than cancer cells transfected with empty plasmid. This result indicated that the skeletal structure of lung adenocarcinoma cells may be rearranged after the transfection of LINC00472. The present study suggested that LINC00472 may be used as a tumor suppressor that affects the mechanical properties of lung adenocarcinoma cells, and the cellular mechanical properties may be used as a potential biomarker. Therefore, LINC00472 may be used as a potential biomarker for lung adenocarcinoma diagnostics and treatment. In conclusion, LINC00472, which is lowly expressed in lung adenocarcinoma tissue, changes the biophysical properties of cells and inhibits the migration and invasion of lung adenocarcinoma cells by binding to YBX1. Further research may find that LINC00472 is a potential biomarker of lung adenocarcinoma.

## Supplementary information

Supplementary Figure 1

Supplementary Figure 2

Supplementary Figure 3

Supplementary Figure 4

Supplementary Figure Legends

Supplementary Table.1

## Data Availability

The RNA-seq data set are shared and can be found in the GEO database with the accession number GSE27262 and GSE31210. Besides, the RNA-Seq dataset of lung adenocarcinoma was downloaded from TCGA data.
