# Supplementary figures and images for: LncRNA LINC00472 regulates cell stiffness and inhibits the migration and invasion of lung adenocarcinoma by binding to YBX1
Source: Cell Death Dis. 2020 Nov 3;11(11):945. doi: 10.1038/s41419-020-03147-9 (PMC7609609; doi:10.1038/s41419-020-03147-9)

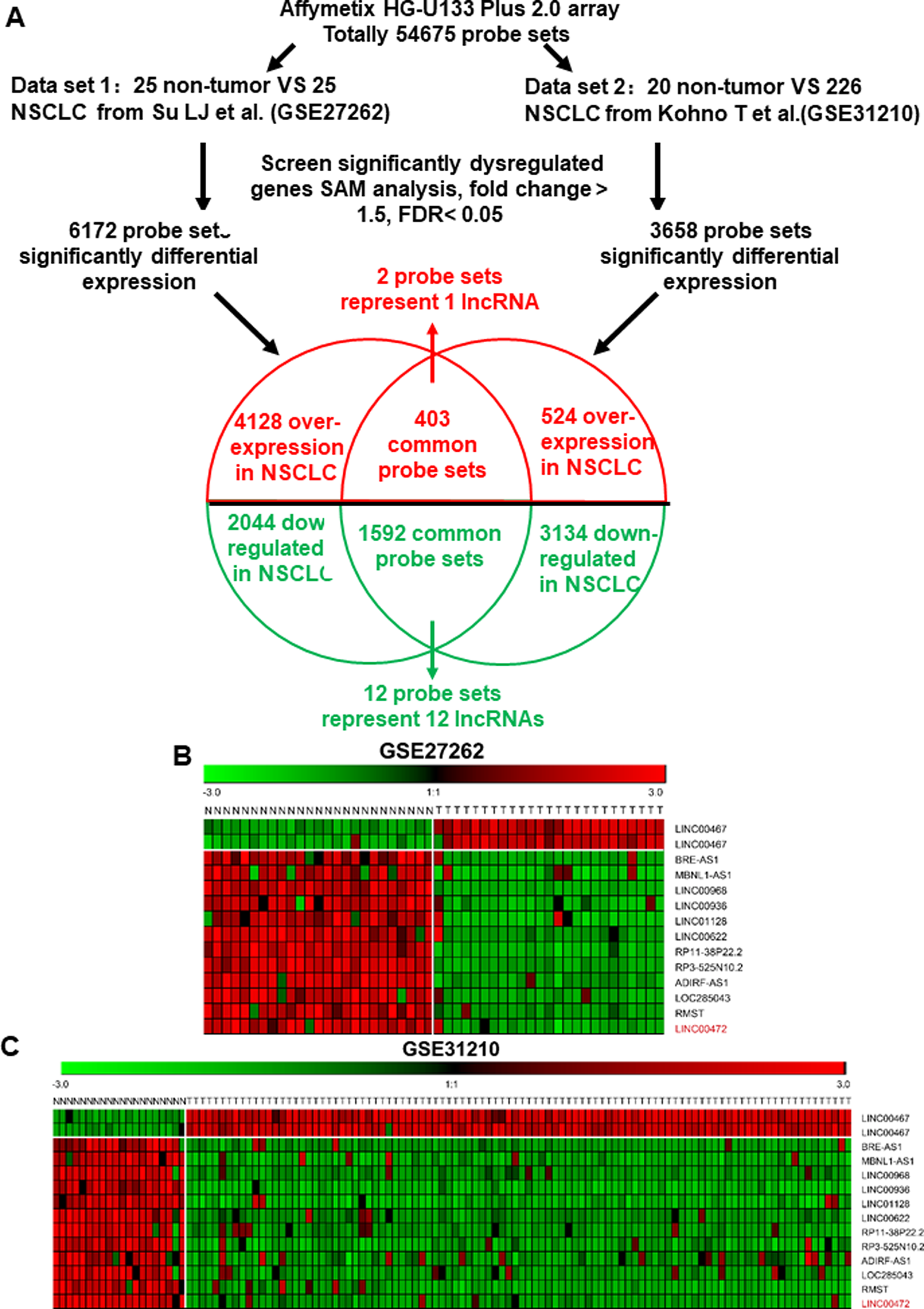

Supplement: Supplementary file 1 — Supplementary Figure 1 [file 41419_2020_3147_MOESM1_ESM.tif]

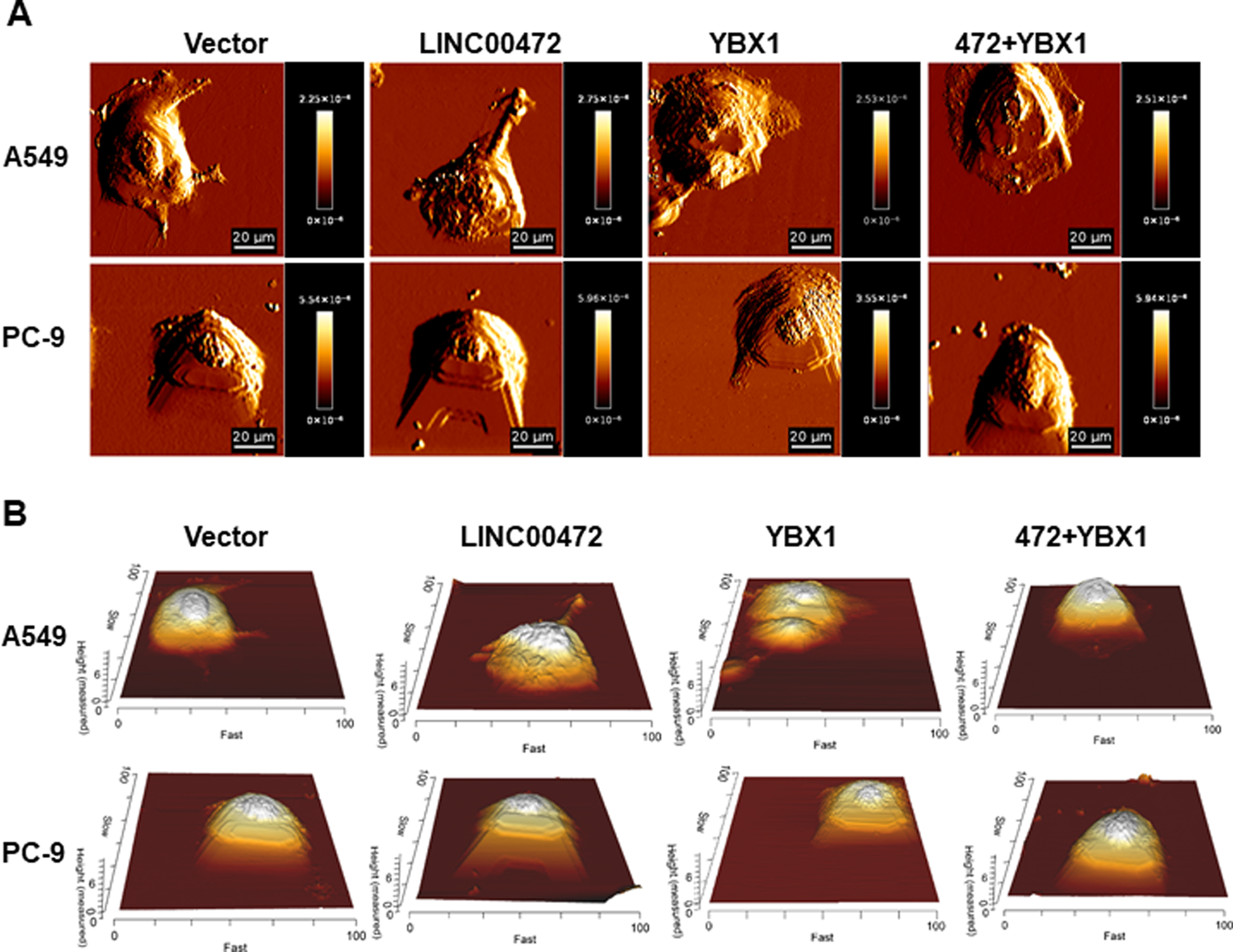

Supplement: Supplementary file 2 — Supplementary Figure 2 [file 41419_2020_3147_MOESM2_ESM.tif]

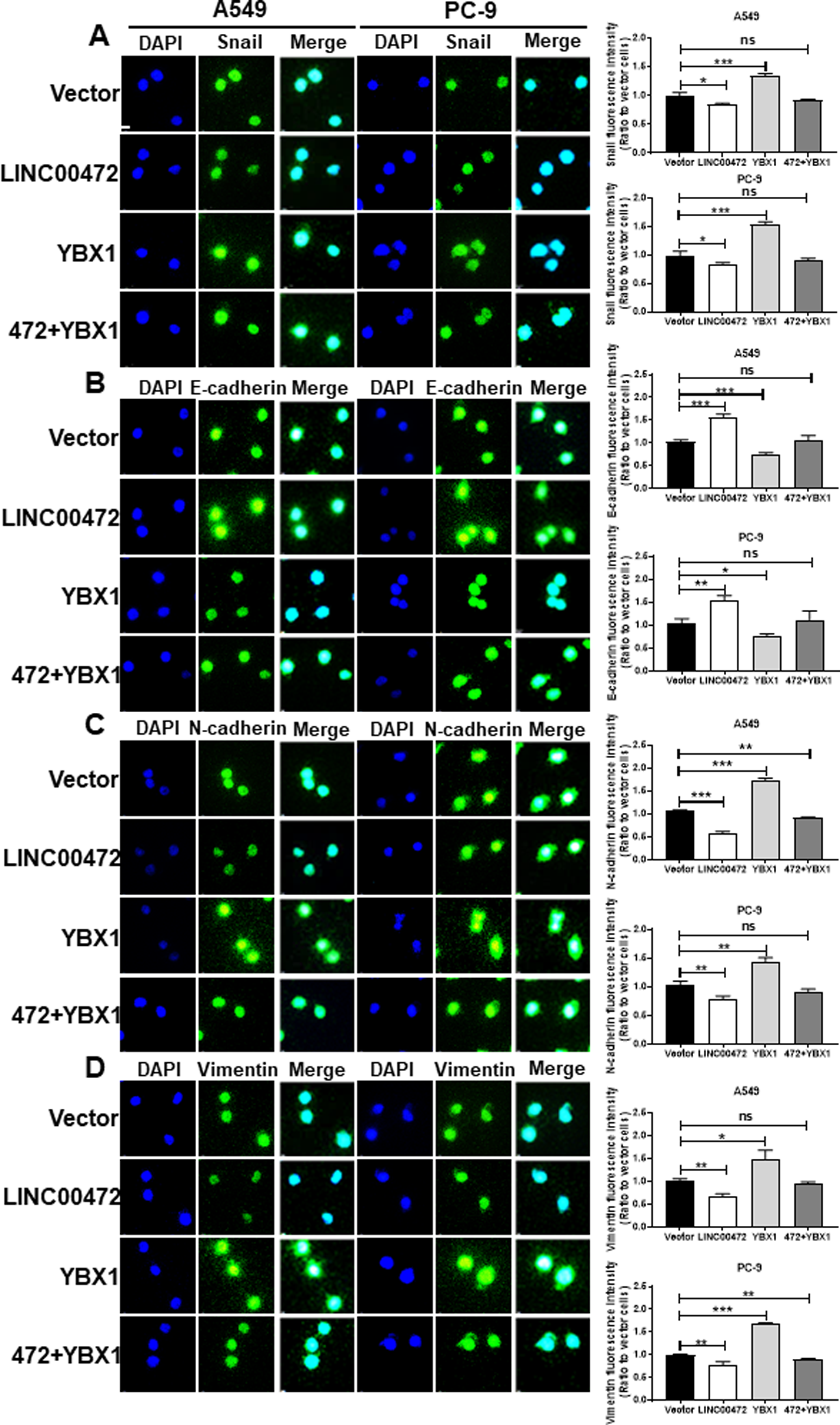

Supplement: Supplementary file 3 — Supplementary Figure 3 [file 41419_2020_3147_MOESM3_ESM.tif]

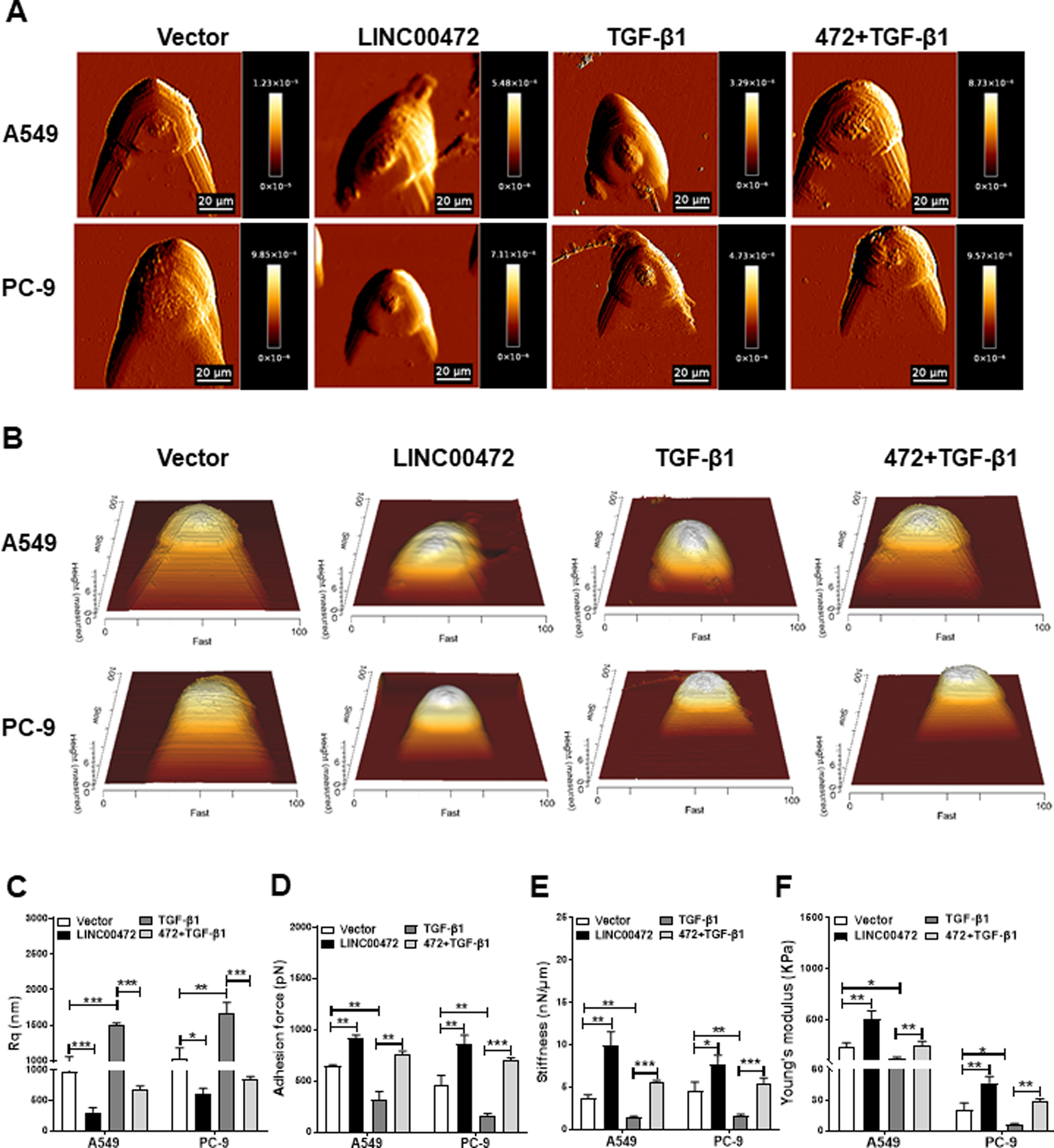

Supplement: Supplementary file 4 — Supplementary Figure 4 [file 41419_2020_3147_MOESM4_ESM.tif]
