# Supplementary material for: LncRNA LINC00472 regulates cell stiffness and inhibits the migration and invasion of lung adenocarcinoma by binding to YBX1
Source: Cell Death Dis. 2020 Nov 3;11(11):945. doi: 10.1038/s41419-020-03147-9 (PMC7609609; doi:10.1038/s41419-020-03147-9)
Supplement: Supplementary file 5 — Supplementary Figure Legends [file 41419_2020_3147_MOESM5_ESM.docx]

**Supplementary Figure 1. Dysregulated lncRNA expression analysis using two GEO datasets. a.** Schematic overview of the workflow used to identify and validate the dysregulated lncRNAs in two lung adenocarcinoma microarray data cohorts. The dysregulated lncRNAs in these GEO datasets are shown in **b** and **c**.

**Supplementary Figure 2. LINC00472 regulates the cell surface morphology through YBX1. a.** Representative AFM deflection images of A549 and PC-9 cells transfected or cotransfected with the LINC00472 overexpression vector or the YBX1 overexpression vector. **b.** 3D distribution of the cell height of A549 and PC-9 cells transfected or cotransfected with the LINC00472 overexpression vector or the YBX1 overexpression vector. Vector: empty vector; LINC00472: LINC00472 overexpression vector; YBX1: YBX1 overexpression vector.

**Supplementary Figure 3. The changes of EMT makers were detected by immunofluorescence.** The protein expression of Snail **(a)**, E-cadherin **(b)**, N-cadherin **(c)** and vimentin **(d)** were detected in A549 and PC-9 cells after transfection or cotransfection with the LINC00472 overexpression vector or the YBX1 overexpression vector. Statistics of fluorescence intensity in A549 and PC-9 cells after the transfection of different vectors. Vector: empty vector；LINC00472: LINC00472 overexpression vector; YBX1: YBX1 overexpression vector. *, *p* < 0.05; **, *p* < 0.01; ***, *p* < 0.001, ns: no significance. Scale bar=19 μm.

**Supplementary Figure 4. Detection of changes in cell biophysical properties after TGF-β1 induction of EMT. a.** Representative AFM deflection images of A549 and PC-9 cells transfected or cotransfected with the LINC00472 overexpression vector or the TGF-β1 overexpression vector. **b.** 3D distribution of the cell height of A549 and PC-9 cells transfected or cotransfected with the LINC00472 overexpression vector or the TGF-β1 overexpression vector. **c.** Analysis of the surface morphology (Rq) of A549 and PC-9 cells. **d.** Analysis of the adhesion of A549 and PC-9 cells. **e.** Analysis of the stiffness of A549 and PC-9 cells. **f.** Analysis of Young's modulus of A549 and PC-9 cells. Vector: empty vector; LINC00472: LINC00472 overexpression vector; TGF-β1: TGF-β1 overexpression vector. *, *p* < 0.05; **, *p* < 0.01; ***, *p* < 0.001.
