# Supplementary material for: LncRNA LINC00472 regulates cell stiffness and inhibits the migration and invasion of lung adenocarcinoma by binding to YBX1
Source: Cell Death Dis. 2020 Nov 3;11(11):945. doi: 10.1038/s41419-020-03147-9 (PMC7609609; doi:10.1038/s41419-020-03147-9)
Supplement: Supplementary file 6 — Supplementary Table.1 [file 41419_2020_3147_MOESM6_ESM.docx]

**Supplementary Table 1. A list of primers used in this study**

| **Primer** | **Sequence** |
| --- | --- |
| LINC00472-F | 5'-GGAGATACGGTCAGAGAGGC-3' |
| LINC00472-R | 5'-GGTGCTTCATAGGGGTCTGT-3' |
| ACTA1-F | 5'-TCACCAACTGGGACGACATG-3' |
| ACTA1-R | 5'-GTCACCGGAGTCCATCACGAT-3' |
| YBX1-F | 5'-TGCAGGGAGAAGTGATGGAG-3' |
| YBX1-R | 5'-TTAGGGTTTTCTGGGCGTCT-3' |
| Snail-F | 5'-CCTCCAGACCCACTCAGATG-3' |
| Snail-R | 5'-CAGTGAGAAGGATGTGGGGT-3' |
| Slug-F | 5'-CTTTTTCTTGCCCTCACTGC-3' |
| Slug-R | 5'-GCTTCGGAGTGAAGAAATGC-3' |
| Twist1-F | 5'-AGTCCGCAGTCTTACGAGGA-3' |
| Twist1-R | 5'-CCAGCTTGAGGGTCTGAATC-3' |
| Twist2-F | 5'-CAGAGCCTTTCCAGCAACTC-3' |
| Twist2-R | 5'-TCGCTCGACTTCTTGCTGTA-3' |
| ZEB1-F | 5'-CAGGGAGGAGCAGTGAAAGA-3' |
| ZEB1-R | 5'-CTCTTCAGGTGCCTCAGGAA-3' |
| ZEB2-F | 5'-AGGAATGACAAGCCCCATCA-3' |
| ZEB2-R | 5'-GAGGGTTACTGTTGGGAGCT-3' |
